# G-Protein Coupled Receptor Dysregulation May Play Roles in Severe Preeclampsia—A Weighted Gene Correlation Network Analysis of Placental Gene Expression Profile

**DOI:** 10.3390/cells11050763

**Published:** 2022-02-22

**Authors:** Manuel S. Vidal, Christian Deo T. Deguit, Gracia Fe B. Yu, Melissa D. Amosco

**Affiliations:** 1 College of Medicine, University of the Philippines, Manila 1000, Philippines; 2 Institute of Human Genetics, National Institutes of Health, University of the Philippines, Manila 1000, Philippines; ctdeguit@up.edu.ph; 3 Department of Biochemistry and Molecular Biology, University of the Philippines Manila, Manila 1000, Philippines; grafebyu@gmail.com; 4 Department of Obstetrics and Gynecology, Philippine General Hospital, University of the Philippines, Manila 1000, Philippines; mdamosco@up.edu.ph

**Keywords:** preeclampsia, G-protein-coupled receptors, GPCR, weighted gene correlation network analysis, WGCNA

## Abstract

Preeclampsia is one of the major hypertensive diseases of pregnancy. Genetic factors contribute to abnormal placentation. The inadequate transformation of cytotrophoblasts causes failure of maternal spiral arteries’ remodeling and results in narrow, atherotic-prone vessels, leading to relative placental ischemia. This study aims to explore the possibility of identifying dysregulated gene networks that may offer a potential target in the possible prevention of preeclampsia. We performed a weighted gene correlated network analysis (WGCNA) on a subset of gene expression profiles of placental tissues from severe preeclamptic pregnancies. We identified a gene module (number of genes = 402, GS = 0.35, *p* = 0.02) enriched for several G-protein-coupled receptor (GPCR)-related genes with significant protein–protein molecular interaction (number of genes = 38, FDR = 0.0007) that may play key roles in preeclampsia. Some genes are noted to play key roles in preeclampsia, including *LPAR4/5, CRLR, NPY, TACR1/2*, and *SFRP4/5*, whose functions generally relate to angiogenesis and vasodilation or vasoconstriction. Other upregulated genes, including olfactory and orexigenic genes, serve limited functions in the disease pathogenesis. Altogether, this study shows the utility of WGCNA in exploring possible new gene targets, and additionally reinforces the feasibility of targeting GPCRs that may offer intervention against development and disease progression among severe preeclampsia patients.

## 1. Introduction

Preeclampsia is one of the major hypertensive diseases of pregnancy, affecting about 2–8% of pregnancies [1]. Hypertensive disorders are predominant pregnancy complications, accounting for about 36.7% of maternal deaths in the Philippines [2]. The disease is characterized by new-onset hypertension beyond 20 weeks of gestation, which may further evolve to involve the hematologic, pulmonary, neurologic, and hepatic systems [1]. The pathogenesis thus has been generalized into two phases, an early defect in placentation and a later maternal syndrome due to an abnormal upregulation of placental antiangiogenic factors [3].

The etiology of preeclampsia remains unclear. Several theories have been proposed, including immunologic, genetic, and environmental factors. More recently, preeclampsia has been categorized into two subtypes depending on the period of onset or recognition of the disease [4]. Early-onset preeclampsia (<34 weeks) has been characterized to progress to relative placental ischemia due to the failure of maternal spiral arteries to remodel into high-capacitance, high-flow vessels [4]. The narrower preeclamptic spiral arteries are prone to atherotic changes, with fibrinoid necrosis, foamy macrophage accumulation, and mononuclear perivascular infiltrations [5]. On the other hand, in late-onset preeclampsia (≥34 weeks), there is no dramatic change in the uteroplacental vasculature and preeclampsia itself stems from maternal stress late in pregnancy, usually associated with co-existing maternal systemic inflammatory conditions [4].

The only definitive treatment for preeclampsia is the timely delivery of the fetus and the placenta. No standard therapy has yet been approved for antepartum disease [6]. Low-dose aspirin (150 mg/day) has been shown to decrease the incidence of preeclampsia in high-risk women, and is now recommended despite its unclear mechanism [1].

As previously mentioned, several genes have been implicated in the development of a diseased placenta. Therefore, we sought to determine dysregulated genes with significant protein–protein interactions in a sample subset of severe preeclampsia patients, and to determine the possible molecular targets for intervention. Sitras et al. previously showed that specific genes involved in Notch-, tumor growth factor-beta (TGFβ)-, and vascular endothelial growth factor (VEGF)-signaling pathways were differentially expressed in severe preeclampsia. However, their analysis was limited to differential gene expression; more effective distinguishers of associated molecular pathways, such as gene correlations, were not evaluated [7]. Weighted correlation network analysis (WGCNA) provides an opportunity to discover multiple networks associated with dysregulations. By determining hub genes that act as central players within the modules, biologically meaningful insights related to the clinical traits of interest can be acquired. 

Accordingly, we opted to re-analyze the placental tissue gene expression data (GSE10588) from the study of Sitras et al. through WGCNA [7]. There is a dearth of studies that utilize WGCNA to discover dysregulated networks in severe preeclampsia, and so this paper aimed to uncover and describe additional molecular pathways important in the pathology of preeclampsia.

## 2. Materials and Methods

### 2.1. Population and Evaluation of Gene Expression

Gene expression profiles were downloaded, comprising of placental tissues from Caucasian patients with normal pregnancy (*n* = 17) and with severe preeclampsia (*n* = 26) from the study by Sitras et al. (2009) from the public functional genomics data repository Gene Expression Omnibus (GEO accession number: GSE10588); 30k Human Genome Survey Microarray v.2.0 (Applied Biosystems, Waltham, MA, USA) was utilized to evaluate the gene expression profiles. Although this study was published in 2009, the last update to the gene expression dataset since then was in 2016 [7]. Severe preeclampsia was defined as either (1) at least 160 mmHg systolic blood pressure and/or 110 mmHg diastolic blood pressure, measured on at least two resting occasions 6 h apart, with proteinuria ≥2+ on dipstick,; or (2) hemolysis, elevated liver enzymes, and low platelet syndrome after 20 weeks of gestation. Exclusion criteria included pregnant women with pre-existing chronic hypertension or gestational hypertension without proteinuria, renal disease, systemic lupus erythematosus, or diabetes [7]. All other phenotypic data of the study population are included in Table S1.

### 2.2. Identification of Possible Molecular Targets through WGCNA

All data preprocessing and integration procedures such as annotation of known genes, collapsing of probes with multiple gene targets, and WGCNA were performed using R statistical programming language (version 3.5.1) (Vienna, Austria) [8]. Briefly, a scale-free topology network of genes was constructed by calculating the Pearson correlation between different pairs of genes in all samples. To calculate the adjacency matrix, the soft-thresholding power β applied to the correlation data was set to 12. The adjacency matrix was transformed into a topological overlap matrix, from which the dissimilarity values were used to construct the scale-free topology network. Gene modules from the resulting network were produced by setting the minimum module size to 100 and using the dynamic hybrid tree cut algorithm by merging modules with eigengene correlation values less than 0.60. Module eigengene (ME) values for each module were then calculated, defined as the weighted average of gene expression for each module. Module membership (MM) for each gene member in a module was also determined as the absolute correlation between the expression of the gene members and the module ME. To identify the gene modules significantly associated with preeclampsia (i.e., gene modules of interest), Gene Significance (GS) values, which correlate with ME and the presence of preeclampsia, were calculated, and a *p*-value of <0.05 was considered statistically significant.

Gene modules of interest were then characterized via functional enrichment analysis to identify the associated molecular pathways. This analysis was performed by uploading the entire gene list of each module to the STRING 11.0 database (https://string-db.org, accessed on 10 March 2020). This online resource can perform gene-set enrichment analysis utilizing recognized classification systems, such as Gene Ontology and Reactome [9]. STRING was also used to generate protein–protein interaction (PPI) networks (required interaction score threshold = 0.9) for the gene modules of interest.

## 3. Results

**Figure 1:**
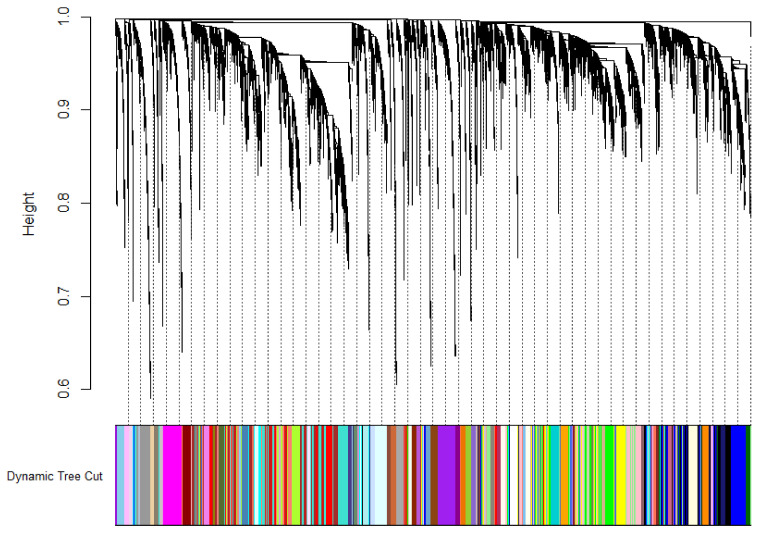
WGCNA results of the placental samples from severe preeclampsia patients display multiple modules of co-expressed genes, assigned by their various module colors.

Twenty-two discrete clusters of strongly correlated genes were identified and assigned individual color schemes. Figure 1 shows the individual modules from the R output, showing differentially expressed gene modules determined using aggregated ME values.

**Figure 2:**
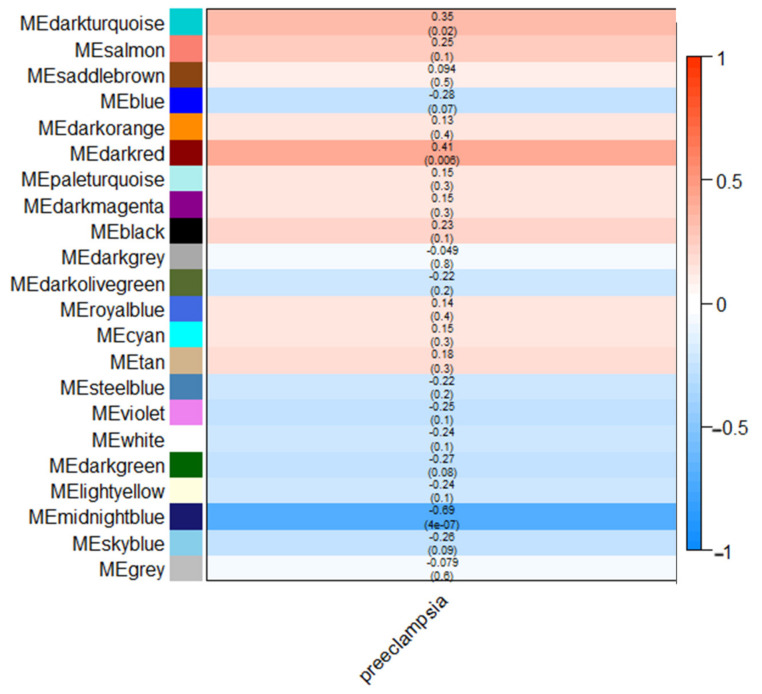
Module–trait relationship heatmap showing correlation of modules with severe preeclampsia. Correlation values (i.e., mean gene significance (GS)) and the corresponding *p*-value from correlation analysis are indicated in each cell; red shading indicates a direct correlation between the expression of the module gene members and the occurrence of severe preeclampsia, while blue shading indicates the inverse correlation.

**Figure 3:**
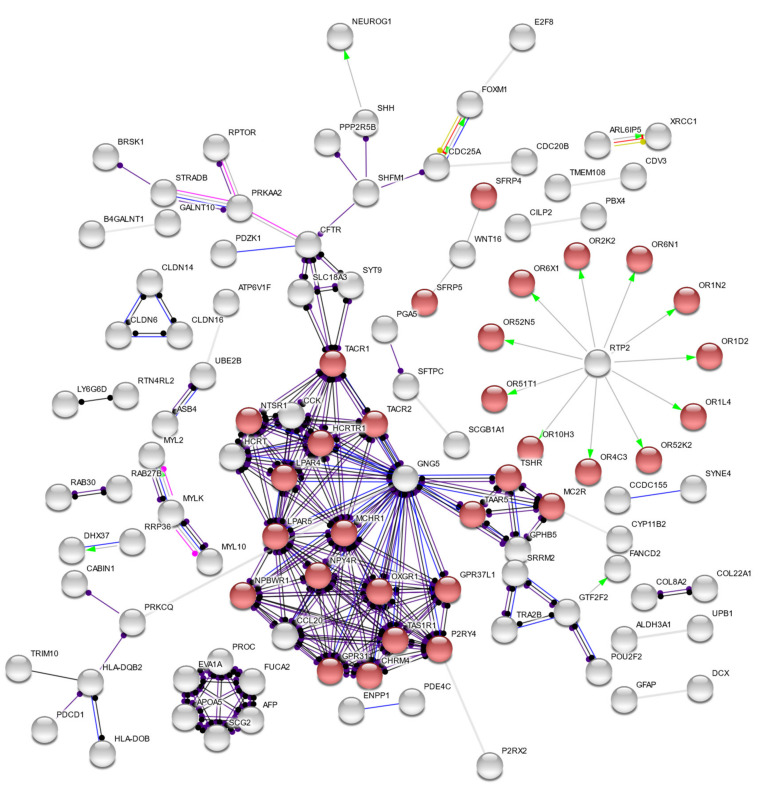
Protein–protein interaction network for this study, showing that the dark turquoise module is comprised of genes involved mostly in receptor activities. Highlighted in red are genes implicated in G-protein-coupled receptor activity, having the lowest false discovery rate (0.0007).

Among all modules, only a handful were found to be correlated with severe preeclampsia—the dark turquoise module (*n* = 402, GS = 0.35, *p* = 0.02), dark red module (*n* = 3162, GS = 0.41, *p* = 0.006), and midnight blue module (*n* = 1340, GS = −0.69, *p* = 4 × 10^−7^). Figure 2 shows a summary of the modules, arranged in decreasing eigengene values. For all three modules, the dark turquoise module contained fewer genes compared to the dark red module and midnight blue module. As the latter two modules would result in a significantly large number of nodes in the resulting networks and cause further confounding, we opted to further analyze only the dark turquoise module for this study; we provide the results of the dark red and midnight blue modules in the Supplementary sections (Supplementary Tables S3 and S4, and Supplementary Figures S1 and S2). Gene enrichment analysis using the STRING database showed that most of the genes involved in the dark turquoise network belonged to the G-protein coupled receptor (GPCR) activities, as shown in Figure 3. Table 1 shows the functional enrichment analysis, while Table 2 lists genes with significant protein–protein molecular function interactions in the dark turquoise module. Individual annotations of GPCR genes are summarized in Table S1.

## 4. Discussion

The active search for a possible cure to preeclampsia is an area of great research interest. Sitras et al. utilized differential gene expression in order to determine possible gene targets for severe preeclampsia [7]; however, uncovering the molecular mechanisms underlying the disease requires a more robust bioinformatic analysis that would entail discovering significant molecular networks that would bring about the preeclamptic phenotype. In this study, we utilized WGCNA for a better noninterventional target exploration as this enables better comparisons between interacting genes instead of simply enumerating differentially expressed genes.

The majority of the upregulated genes in this study belong to the GPCR family, as seen in Figure 3 and Table 2. GPCRs have recently been implicated as potential players in the pathogenesis of preeclampsia; some have been identified to be elevated in the course of normal pregnancy and decreased in preeclamptic patients [10]. Here, we provide additional evidence that dysregulations in the GPCR profile among severe preeclampsia patients may contribute to disease progression. The GPCR activity-related genes within the dark turquoise module may also play roles in preeclampsia, as seen in Table S2.

Notable of the 47 GPCR-related genes would be the genes encoding for the lysophosphatidic acid receptors (LPAR), *LPAR4* and *LPAR5*. Lysophosphatidic acid (LPA), a product of the plasma enzyme autotaxin (ATX), exerts signaling effects via binding to its cognate receptors, LPAR1–6 [11,12]. In preeclamptic patients, interestingly, the mRNA expression levels of LPAR1–5 have been shown to be elevated in placental tissues compared to those of normal pregnant women; our findings corroborate upregulation of *LPAR4* and *LPAR5* [13]. There is a concomitant decrease in ATX production in preeclamptic patients, which has been demonstrated to occur in early-onset hypertensive diseases of pregnancy [14]. Reductions in ATX production, which translates to a reduction in LPA concentrations, in the preeclamptic placenta are involved in impaired vascular remodeling. Therefore, increasing the LPAR levels could be a compensatory action by the placenta to boost downstream Wnt activation to maintain baseline function. However, future experiments are warranted to demonstrate this hypothesis. In vivo, however, LPAR3 seems to be the only protein expressed in the placenta; however, contributions of LPAR4 and LPAR5 outside the placenta may not be dismissed [13]. LPAR4 inhibition has been demonstrated to decrease the experimental atherosclerosis elicited by adeno-associated virus expressing gain-of-function allele of the PCSK9 D377Y mutation in rats fed a fat-rich diet [15]. LPA binding to LPAR5 may result in platelet activation, and inhibition of LPAR5 activity attenuates platelet activation in vitro [16]. Therefore, the upregulations we observed in this study may be related to dysregulations outside the fetomaternal unit that may contribute to overall preeclampsia pathogenesis. Previous studies were also able to synthesize receptor-specific agonists and antagonists specific for the different LPAR subtypes [17,18]. However, these compounds are yet to be utilized for in vivo studies in diseases involving the LPARs, such as preeclampsia.

The calcitonin receptor-like receptor (CRLR) and its endogenous nonallelic vasodilatory peptide ligands, calcitonin gene-related peptide (CGRP) and adrenomedullin, mediate vasodilation in the placental endothelial and vascular smooth muscle compartments [19]. CRLR has been demonstrated to be upregulated in preeclamptic patients without changes to its native ligands in mild preeclampsia; concomitant activation of CGRP expression may increase vasodilation in the hypoxic placenta [20]. Our findings corroborate this upregulation in severe preeclampsia patients. Nitric oxide deficiency has been demonstrated to function as a switch that triggers an increase in *CRLR* expression in preeclamptic patients [21]. However, there seems to be a contradictory finding in an earlier study that found that its levels were instead downregulated in placental tissue [22]. There might be differential placental gene expression depending on the severity of preeclampsia, as seen in this study, or depending on the timing of when this gene is observed. Nonetheless, it is important to note that this gene is dysregulated in preeclampsia, and further definitive studies are needed to verify the status of its regulation in preeclampsia.

Systemic levels of neuropeptide Y (NPY), a peptide that acts as a sympathetic activator and vasoconstrictor, were found to be elevated in preeclampsia patients as compared to the normal pregnancy population [23,24]. The authors posit that decreases in the expression of NPY receptors probably leads to decreased angiogenesis and vasodilation [25]. It is plausible that NPY may function through upregulated levels of the neuropeptide Y-4 receptor (*NPY4R*) to induce vasoconstriction, although future studies need to look into the actual expression of this receptor among preeclamptic patients.

Substance P and substance K are two neurokinins that are implicated in various physiological functions, but their role in preeclampsia has not yet been established. These substances act on their specific receptors, encoded by the genes tachykinin receptor (TACR) 1 (*TACR1*) and *TACR2*, respectively. Substance P and substance K are involved in vasodilation and causes subsequent falls in blood pressure [26]. Substance P has also been shown to be upregulated following tissue injury in order to regenerate blood vessels [27]. In the context of preeclampsia, these genes encoding for tachykinin receptors may be upregulated as a compensatory mechanism in order to alleviate hypertension, although mechanistic studies are required to validate this hypothesis.

Secreted Frizzled-related proteins (SFRPs), another signaling component family, are the largest group of Wnt/β-catenin pathway inhibitors [28]. This inhibition causes eventual degradation of β-catenin, which leads to purported apoptosis of target tissues [29,30,31]. In particular, the SFRPs are suggested to interfere with the Wnt signal as a dominant negative mechanism [29]. Frizzled proteins may also affect angiogenesis in placental tissues, as Wnt–Fzd5 signaling has been shown to upregulate VEGF expression as well as the vascularization of primary villi [32]. *SFRP4* and *SFRP5* are observed to be upregulated in patients with severe preeclampsia [32,33]. It is suggested that a decrease in Wnt2 pathway expression in the placenta via *SFRP4* expression and inhibition may play a role in the pathophysiology of disease [31]. Interestingly, SFRP5 expression seems to be absent in the placenta in immunohistochemistry studies [33]. Therefore, it is plausible that, despite the upregulation in mRNA transcription seen in this study, *SFRP5* expression is repressed post-transcriptionally; more studies are needed to verify this observation.

Other upregulated genes that we found to have significant protein–protein interactions do not have clear physiological roles yet, or limited functions in preeclampsia and/or pregnancy at best. These genes include brain-specific angiogenesis inhibitor 3 (*BAI3*), sortilin-related VPS10 domain-containing receptor 2 (*SORCS2),* trace amine-associated receptor 5 *(TAAR5*), and a number of G-protein-coupled receptors (GPRs) (*GPR113*)*, GPR31, GPR37L1,* and *GPRC5D*). One gene of interest is cholinergic receptor muscarinic 4 (*CHRM4*), a choline metabolism gene that is negatively correlated with circulating soluble factor fms-like tyrosine kinase-1 (sFLT1) concentrations; it is suggested that *CHRM4* may play an as yet unknown regulatory role in sFLT1 production and preeclampsia pathogenesis [34]. Interestingly, one cluster of genes found to be significantly upregulated included a number of olfactory receptors (OR) (*OR10H3, OR1D2, OR1L4, OR1N2, OR2K2, OR4C3, OR51T1, OR52K2, OR52N5, OR6N1,* and *OR6X1*). No putative mechanisms have been put forth for this unusual phenomenon. It has been reported that, in the chorion portion of the fetal membranes of cases of severe preeclampsia, some olfactory receptors were upregulated compared to normal pregnancies, different from the receptors reported in this paper [35]. Although “olfactory hypersensitivity” is a known phenomenon in pregnancy or even in preeclampsia, we cannot draw any conclusions about it due to the lack of studies [36].

It would also be prudent for future studies to look into genes with significant protein–protein interactions in the dark red and midnight blue modules. As found in our Supplementary tables, these modules are enriched with functions for RNA binding and protein binding, respectively. Interestingly, immune activation in preeclampsia has been hypothesized to be, in part, caused by cell-free nucleic acids from the placenta that may bind to various Toll-like receptors (TLRs) that lead to inflammation [37]. Protein binding functions may refer to proteins such as calretinin and heparin-binding epidermal growth factor, which bind target proteins together; we hypothesize that these dysregulations have a greater effect in severe preeclampsia [38].

There are only a few studies utilizing WGCNA in the analysis of genetic predispositions toward preeclampsia. The majority of the studies in this field mainly identify gene polymorphisms in various population groups [39,40,41,42]. We reiterate that WGCNA might be a more suitable analysis compared to the analysis of differential expression of genes for a broader look into the networks responsible for the pathophysiology of the disease. Additionally, as this paper clearly focuses on the Caucasian population, the aforementioned changes may not be applicable to the general population. For instance, an analysis of multiple microarray studies in preeclampsia identified hub genes enriched in the hypoxia-inducible factor 1 (HIF-1) pathway, glutathione metabolism, and placental development [43]. Another study utilizing another group of multiple preeclampsia gene sets showed mainly immune-related genes (bone morphogenetic protein 5 (*BMP5*), cell surface glycoprotein (CD) 200R1 (*CD200R1*) and 28 (*CD28*), and TLR7) that are differentially expressed in preeclampsia [44].

Additional limitations of the study are that (1) the study population does not include a subset of patients with no proteinuria but with severe features; (2) this study generalizes severe preeclampsia gene dysregulations and does not differentiate between early- and late-onset severe preeclampsia, as well as gene predispositions that may be triggered <20 weeks of gestational age; (3) as the initial study by Sitras et al. [7] only utilized microarray studies, we are unable to do a comparative study that also uses protein levels to verify our findings at a translational level; (4) based on phenotypic data, the gestational ages between the two patient groups are significantly different (238 ± 25 weeks vs. 277 ± 9 weeks for severe preeclampsia vs. the control group), and so the initial study may have inadvertent associated changes in terms of placental gene expression that the authors cannot control for; and (5) concomitant treatment with medications such as magnesium sulfate or antihypertensive drugs may have affected the baseline gene expression and no statistical analyses were done to control for this extraneous factor. Future studies could address these five issues for a more robust look into the genetic pathophysiology of preeclampsia. Additionally, the higher incidence of preeclampsia among other ethnic groups, such as Asians and African Americans, warrants similar analyses on a larger scale in order to discover trends that may point to ethnicity-specific genes that may serve as diagnostic and prognostic markers as well as interventional points for preeclampsia.

Moreover, other publicly available gene expression datasets focusing on preeclampsia from the GEO database may be subjected to WGCNA to validate whether the modules we observed are conserved across different gene expression analysis platforms, preeclampsia clinical criteria, and tissue sources. Merging and normalization procedures can also be done on the different datasets to perform a more robust WGCNA that may point to more biologically significant pathways. Nonetheless, this study provides an example of the utilization of WGCNA to identify other genetic dysregulations that can be utilized in theoretical target modeling or experimental intervention studies.

## 5. Conclusions

Using WGCNA, this paper posits the role of dysregulated GPCRs in severe preeclampsia, in addition to aberrantly expressed genes in the established study by Sitras et al. [7]. Future research targeting these set of upregulated genes in severe preeclampsia must keep in mind that off-targets may result due to the ubiquitous nature of GPCRs (i.e., some genes may also be present in other organs) and that interventions must be tailored in order to specifically interfere with the relevant preeclampsia pathways. Targeting the lysophosphatidic pathway is an attractive option since LPARs act as receptors commonly activated by ATX-produced LPA, which is correspondingly expressed as an angiogenic factor for placental blood vessel formation. Similarly, targeting the CRLR and NPY pathways may also be therapeutic, since aberrant expression of both genes may be additive towards impaired LPAR expression, which leads to eventual vasoconstriction and hypoxia within endothelial cells, either in the systemic vasculature or within the placental blood supply. This study also establishes the need for a re-examination of public datasets in which WGCNA can be done in order to determine significant protein–protein interactions that may be relevant to disease progression.

## Figures and Tables

**Table 1:** Selected differentially expressed gene modules, grouped according to decreasing eigengene values.

Module Color	GS Value	Number of Genes	Top Five Module Genes
Dark red	0.41 (*p* = 0.006)	3162	*MRPL19, FLJ12903, C2orf33, SLC25A5, ZNF627*
Dark turquoise	0.35 (*p* = 0.02)	402	*GPR92, GPR7, PADI3, MATN4, BAZ2B*
Midnight blue	−0.69 (*p* = 4 × 10^−7^)	1340	*HKE2, BANF1, CAPNS1, CREBL1, C6orf109*

**Table 2:** Summary of genes with significant protein–protein molecular function interactions in the dark turquoise module, implicating genes involved mostly in receptor activities. The G-protein-coupled receptor activity network displayed the lowest false discovery rate (0.0007).

#Term ID	Description	Observed Gene Count	Background Gene Count	False Discovery Rate
GO:0004930	G-protein-coupled receptor activity	38	824	0.0007
GO:0008188	neuropeptide receptor activity	8	47	0.003
GO:0004888	transmembrane signaling receptor activity	45	1226	0.0044
GO:0008528	G-protein-coupled peptide receptor activity	12	132	0.0044
GO:0060089	molecular transducer activity	50	1483	0.0105
GO:0038023	signaling receptor activity	48	1429	0.0137

## Data Availability

Not applicable.

## Supplementary Materials

The following supporting information can be downloaded at: https://www.mdpi.com/article/10.3390/cells11050763/s1: Table S1. Phenotypic data for the patients included in the RNA microarray studies by Sitras et al. [7]. Table S2. Dark turquoise module genes related to G-protein-coupled receptor activity, and their annotated functions. Table S3. Summary of genes with significant protein–protein molecular function interactions in the dark red module. Proteins involved in RNA binding have the lowest false discovery rate (0.00098). Table S4. Summary of genes with significant protein–protein molecular function interactions in the midnight blue module. Proteins involved in protein binding have the lowest false discovery rate (0.00043). Figure S1. Protein–protein interaction network for the dark red module. The figure shows some of the connected nodes from the 1419 total nodes. Figure S2. Summary of genes with significant protein–protein molecular function interactions in the midnight blue module. The figure shows some of the connected nodes from the 1262 total nodes.
