# Supplementary material for: G-Protein Coupled Receptor Dysregulation May Play Roles in Severe Preeclampsia—A Weighted Gene Correlation Network Analysis of Placental Gene Expression Profile
Source: Cells. 2022 Feb 22;11(5):763. doi: 10.3390/cells11050763 (PMC8909297; doi:10.3390/cells11050763)
Supplement: Supplementary file 1 [file cells-11-00763-s001.zip › cells-1559673-supplementary.pdf]

Table S1. Phenotypic data for the patients included in the RNA microarray studies in Sitras et al. (2009).

Parameter	Severe preeclampsia (n = 16)	Healthy controls (n = 21)	p
Maternal age (years)	30.5 ± 5.2	30.2 ± 4.8	0.9
Nulliparous n (%)	7 (44%)	9 (43%)	1
Body Mass Index at first antenatal visit (kg/m2)	25.9 ± 4.8	24.8 ± 5.3	0.5
Body Mass Index before delivery (kg/m2)	31.2 ± 5.8	29.8 ± 4.2	0.5
Mean arterial pressure before delivery (mmHg)	132 ± 10	89 ± 8	<0.0001
Proteinuria (g/l)	3.93 ± 2.5	-	-
Uterine artery pulsatility index (mean of the left and right side)	1.37 ± 0.70	0.74 ± 0.30	0.002
Uterine artery protodiastolic notching	7 (44%)	1 (5%)	0.004
Total uterine artery volume blood flow (ml/min)	458 ± 383	905 ± 572	0.03
Middle cerebral artery pulsatility index	1.50 ± 0.41	1.27 ± 0.30	0.072
Umbilical artery pulsatility index	1.17 ± 0.36	0.78 ± 0.18	0.001
Umbilical vein volume blood flow normalised for birthweight (ml/min/kg)	84.6 ± 47.5	66.2 ± 31.4	0.2
Gestational age at delivery (days)	238 ± 25	277 ± 9	<0.001
Caesarean section	11 (69%)	8 (38%)	0.065
Birthweight (g)	2181 ± 998	3653 ± 619	0.000
Birthweight 10 percentile	7 (44%)	3 (14%)	0.046
Placental weight (g)	445 ± 183	648 ± 154	0.001
5 min APGAR score	8-10	9-10	0.1
Arterial cord blood pH	7.26 ± 0.07	7.26 ± 0.03	0.9
Arterial cord blood Base Excess	-2.3 ± 3.8	-2.4 ± 1.9	0.9
Venous cord blood pH	7.30 ± 0.05	7.33 ± 0.04	0.1
Venous cord blood Base Excess	-2.00 ± 3.6	-2.23 ± 4.9	0.9

Supplementary Table S2. Darkturquoise module genes related to G protein-coupled receptor activity, and their annotated functions.

BAI3	This p53-target gene encodes a brain-specific angiogenesis inhibitor, a seven-span transmembrane protein, and is thought to be a member of the secretin receptor family. Brain-specific angiogenesis proteins BAI2 and BAI3 are similar to BAI1 in structure, have similar tissue specificities, and may also play a role in angiogenesis.

CHRM4	Muscarinic acetylcholine receptor M4; The muscarinic acetylcholine receptor mediates various cellular responses, including inhibition of adenylate cyclase, breakdown of phosphoinositides and modulation of potassium channels through the action of G proteins. Primary transducing effect is inhibition of adenylate cyclase; Cholinergic receptors muscarinic
EMR3	This gene encodes a member of the class B seven-span transmembrane (TM7) receptor family expressed predominantly by cells of the immune system. Family members are characterized by an extended extracellular region with a variable number of N-terminal epidermal growth factor (EGF)-like domains coupled to a TM7 domain via a mucin-like spacer domain. This gene is closely linked to the gene encoding egf-like molecule containing mucin-like hormone receptor 2 on chromosome 19. This protein may play a role in myeloid-myeloid interactions during immune and inflammatory responses.
GPR113	ADGRF3 (Adhesion G Protein-Coupled Receptor F3) is a Protein Coding gene. Gene Ontology (GO) annotations related to this gene include *G protein-coupled receptor activity* and *transmembrane signaling receptor activity*.
GPR31	12-(S)-hydroxy-5,8,10,14-eicosatetraenoic acid receptor; High-affinity receptor for 12-(S)-hydroxy-5,8,10,14- eicosatetraenoic acid (12-S-HETE). 12-(S)-HETE is an arachidonic acid metabolite secreted by platelets and tumor cells, and known to induce endothelial cells retraction allowing invasive cell access to the subendothelial matrix, which is a critical step for extravasation or metastasis. Ligand-binding lead to activation of ERK1/2 (MAPK3/MAPK1), MEK, and NF-kappa-B; G protein-coupled receptors, Class A orphans
GPR37L1	Prosaposin receptor GPR37L1; Receptor for the neuroprotective and glioprotective factor prosaposin. Ligand binding induces endocytosis, followed by an ERK phosphorylation cascade; G protein-coupled receptors, Class A orphans
GPRC5D	The protein encoded by this gene is a member of the G protein-coupled receptor family; however, the specific function of this gene has not yet been determined.
HCRTR1	Orexin receptor type 1; Moderately selective excitatory receptor for orexin-A and, with a lower affinity, for orexin-B neuropeptide. Triggers an increase in cytoplasmic Ca(2+) levels in response to orexin-A binding; Belongs to the G-protein coupled receptor 1 family
LGR4	The protein encoded by this gene is a G-protein coupled receptor that binds R-spondins and activates the Wnt signaling pathway. This Wnt signaling pathway activation is necessary for proper development of many organs of the body.
LPAR4	Lysophosphatidic acid receptor 4; Receptor for lysophosphatidic acid (LPA), a mediator of diverse cellular activities. Transduces a signal by increasing the intracellular calcium ions and by stimulating adenylyl cyclase activity.
LPAR5	Lysophosphatidic acid receptor 5; Receptor for lysophosphatidic acid (LPA), a mediator of diverse cellular activities

MC2R	Adrenocorticotropic hormone receptor; Receptor for corticotropin (ACTH). This receptor is mediated by G proteins (G(s)) which activate adenylate cyclase (cAMP); Belongs to the G-protein coupled receptor 1 family
MCHR1	Melanin-concentrating hormone receptor 1; Receptor for melanin-concentrating hormone, coupled to both G proteins that inhibit adenylyl cyclase and G proteins that activate phosphoinositide hydrolysis; Belongs to the G-protein coupled receptor 1 family
NLRP6	The protein encoded by this gene binds arginine-vasopressin and may be involved in the arginine-vasopressin-mediated regulation of renal salt-water balance. The encoded protein also mediates inflammatory responses in the colon to allow recovery from intestinal epithelial damage and protects against tumorigenesis and the development of colitis. Finally, this protein can increase activation of NF-kappa-B, activation of CASP1 through interaction with ASC, and cAMP accumulation.
NPBWR1	Neuropeptides B/W receptor type 1; Interacts specifically with a number of opioid ligands. Receptor for neuropeptides B and W, which may be involved in neuroendocrine system regulation, food intake and the organization of other signals. Has a higher affinity for neuropeptide B
NPY4R	Neuropeptide Y receptor type 4; Receptor for neuropeptide Y and peptide YY.
NTSR1	Neurotensin receptor type 1; G-protein coupled receptor for the tridecapeptide neurotensin (NTS). Signaling is effected via G proteins that activate a phosphatidylinositol-calcium second messenger system. Signaling leads to the activation of downstream MAP kinases and protects cells against apoptosis
OR10H3	Olfactory receptor 10H3; Odorant receptor; Olfactory receptors, family 10
OR1D2	Olfactory receptor 1D2; Odorant receptor which may be involved in sperm chemotaxis. Bourgeonal is a strong chemoattractant for sperm in vitro and is shown to be a strong agonist for OR1D2 in vitro.
OR1L4	Olfactory receptor 1L4; Odorant receptor; Olfactory receptors, family 1
OR1N2	Olfactory receptor 1N2; Odorant receptor; Olfactory receptors, family 1
OR2K2	Olfactory receptor 52K2; Odorant receptor; Olfactory receptors, family 52
OR4C3	Olfactory receptor 4C3; Odorant receptor; Olfactory receptors, family 4
OR51T1	Olfactory receptor 51T1; Odorant receptor; Olfactory receptors, family 51
OR52K2	Olfactory receptor 2K2; Odorant receptor; Olfactory receptors, family 2
OR52N5	Olfactory receptor 52N5; Odorant receptor; Olfactory receptors, family 52
OR6N1	Olfactory receptor 6N1; Odorant receptor; Olfactory receptors, family 6
OR6X1	Olfactory receptor 6X1; Odorant receptor; Olfactory receptors, family 6
OXGR1	This gene encodes a G protein-coupled receptor (GPCR) that belongs to the oxoglutarate receptor family within the GPCR superfamily. The encoded protein is activated by the citric acid intermediate, oxoglutarate, as well as several cysteinyl leukotrienes, including leukotrienes E4, C4 and D4, which are implicated in many inflammatory disorders. In mice, a knock-out of this gene leads to middle ear inflammation, changes in the mucosal epithelium, and an increase in fluid behind the eardrum, and is associated with hearing loss.

P2RY4	P2Y purinoceptor 4; Receptor for UTP and UDP coupled to G-proteins that activate a phosphatidylinositol-calcium second messenger system. Not activated by ATP or ADP
SFRP4	Secreted frizzled-related protein 4; Soluble frizzled-related proteins (sFRPS) function as modulators of Wnt signaling through direct interaction with Wnts. They have a role in regulating cell growth and differentiation in specific cell types (By similarity). SFRP4 plays a role in bone morphogenesis. May also act as a regulator of adult uterine morphology and function. May also increase apoptosis during ovulation possibly through modulation of FZ1/FZ4/WNT4 signaling (By similarity). Has phosphaturic effects by specifically inhibiting sodium-dependent phosphate uptake
SFRP5	Secreted frizzled-related protein 5; Soluble frizzled-related proteins (sFRPS) function as modulators of Wnt signaling through direct interaction with Wnts. They have a role in regulating cell growth and differentiation in specific cell types. SFRP5 may be involved in determining the polarity of photoreceptor, and perhaps, other cells in the retina
SORCS2	This gene encodes one family member of vacuolar protein sorting 10 (VPS10) domain-containing receptor proteins. The VPS10 domain name comes from the yeast carboxypeptidase Y sorting receptor Vps10 protein. Members of this gene family are large with many exons but the CDS lengths are usually less than 3700 nt. Very large introns typically separate the exons encoding the VPS10 domain; the remaining exons are separated by much smaller-sized introns. These genes are strongly expressed in the central nervous system.
TAAR5	Trace amine-associated receptor 5; Olfactory receptor specific for trimethylamine, a trace amine. Also activated at lower level by dimethylethylamine. Trimethylamine is a bacterial metabolite found in some animal odors, and to humans it is a repulsive odor associated with bad breath and spoiled food. This receptor is probably mediated by the G(s)-class of G-proteins which activate adenylate cyclase
TACR1	Substance-P receptor; This is a receptor for the tachykinin neuropeptide substance P. It is probably associated with G proteins that activate a phosphatidylinositol-calcium second messenger system. The rank order of affinity of this receptor to tachykinins is: substance P > substance K > neuromedin-K
TACR2	Substance-K receptor; This is a receptor for the tachykinin neuropeptide substance K (neurokinin A). It is associated with G proteins that activate a phosphatidylinositol-calcium second messenger system. The rank order of affinity of this receptor to tachykinins is: substance K > neuromedin-K > substance P
TAS1R1	The protein encoded by this gene is a G protein-coupled receptor and is a component of the heterodimeric amino acid taste receptor T1R1+3. The T1R1+3 receptor responds to L-amino acids but not to D-enantiomers or other compounds. Most amino acids that are perceived as sweet activate T1R1+3, and

	this activation is strictly dependent on an intact T1R1+3 heterodimer. Multiple transcript variants encoding different isoforms have been found for this gene.
TSHR	Thyrotropin receptor; Receptor for the thyroid-stimulating hormone (TSH) or thyrotropin. Also acts as a receptor for the heterodimeric glycoprotein hormone (GPHA2:GPHB5) or thyrostimulin. The activity of this receptor is mediated by G proteins which activate adenylate cyclase. Plays a central role in controlling thyroid cell metabolism (By similarity); Belongs to the G-protein coupled receptor 1 family. FSH/LSH/TSH subfamily

Supplementary Table S3. Summary of genes with significant protein-protein molecular function interactions in the darkred module. Proteins involved in RNA binding have the lowest false discovery rate (0.00098).

#TERM ID	TERM DESCRIPTION	OBSERVED GENE COUNT	BACKGROUND GENE COUNT	FALSE DISCOVERY RATE
GO:0003723	RNA binding	179	1649	0.00098
GO:0003676	Nucleic acid binding	355	3947	0.0294
GO:0003735	Structural constituent of ribosome	29	159	0.0452

Supplementary Table S4. Summary of genes with significant protein-protein molecular function interactions in the midnightblue module. Proteins involved in protein binding have the lowest false discovery rate (0.00043).

#TERM ID	TERM DESCRIPTION	OBSERVED GENE COUNT	BACKGROUND GENE COUNT	FALSE DISCOVERY RATE
GO:0005515	Protein binding	547	7026	0.00043
GO:0005488	Binding	893	12516	0.0011
GO:0042802	Identical protein binding	170	1896	0.0312
GO:0019899	Enzyme binding	194	2239	0.0379

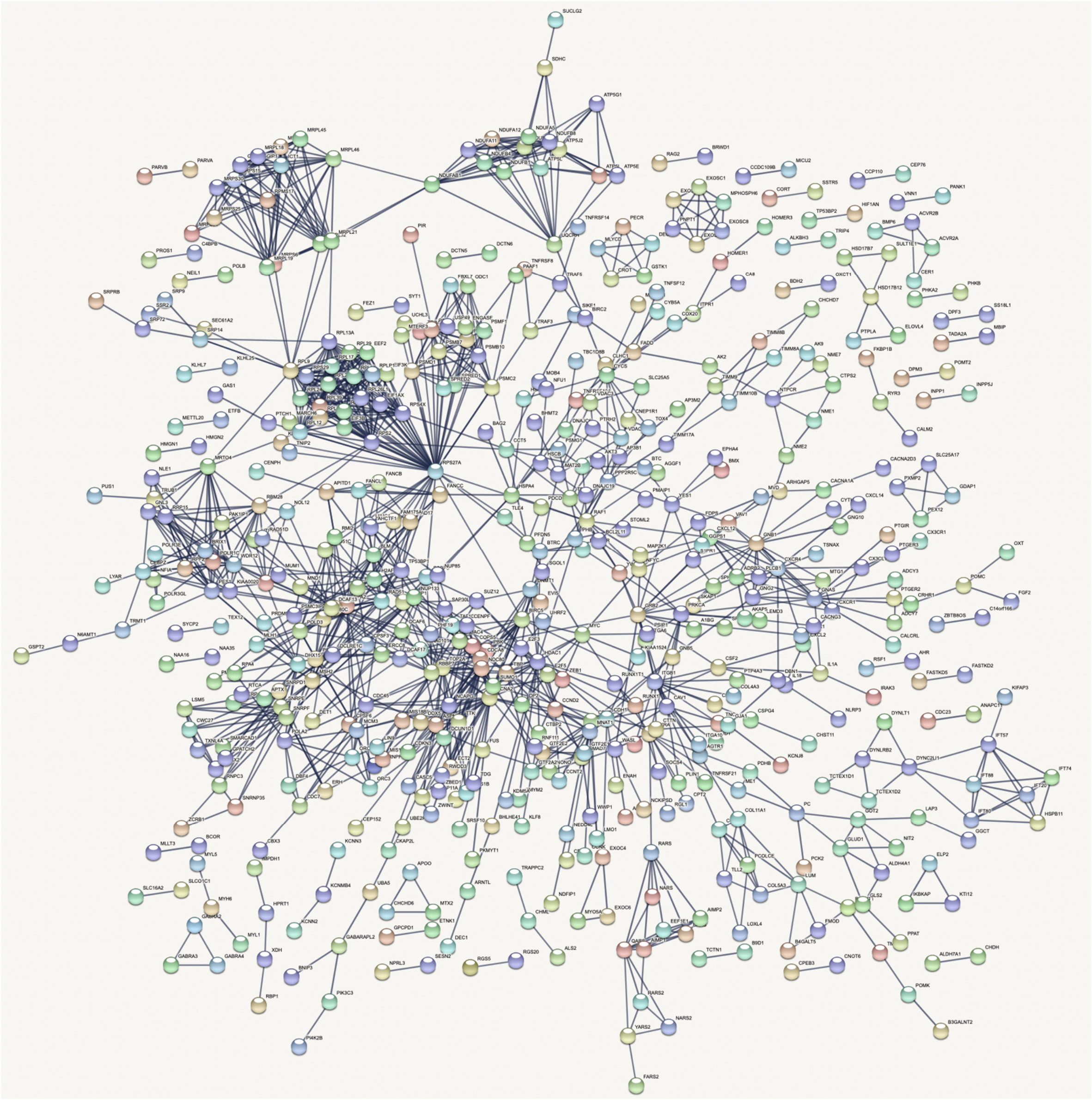

Supplementary Figure S1. Protein-protein interaction network for the darkred module. The figure shows some of the connected nodes from the 1419 total nodes.

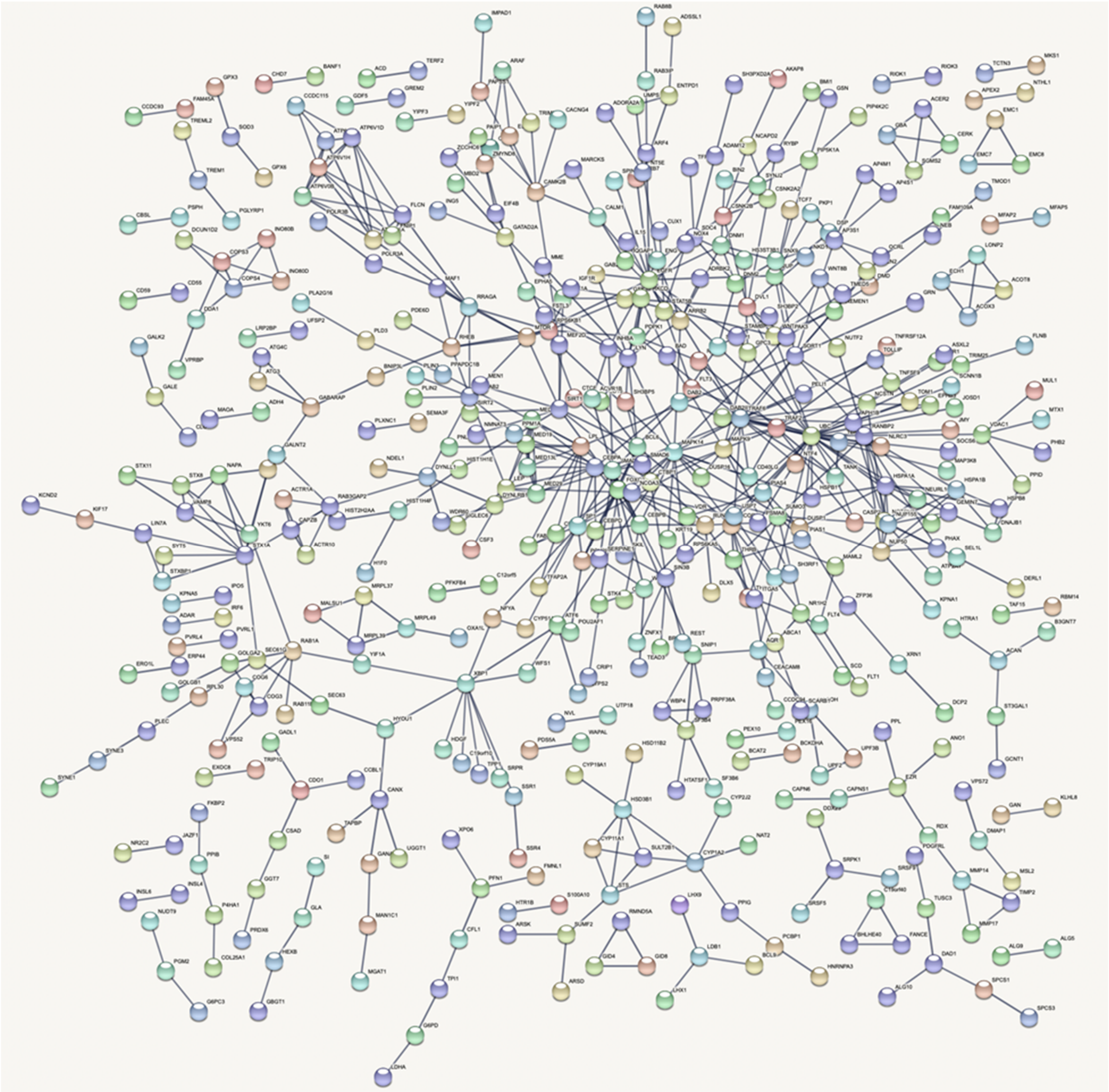

Supplementary Figure S2. Summary of genes with significant protein-protein molecular function interactions in the midnightblue module. The figure shows some of the connected nodes from the 1262 total nodes.
